# Effect of overground gait training with ‘Mobility Assisted Robotic System-MARS’ on gait parameters in patients with stroke: a pre-post study

**DOI:** 10.1186/s12883-023-03357-6

**Published:** 2023-08-09

**Authors:** Anupam Gupta, Navin B. Prakash, Gourav Sannyasi, Faiz Mohamad, Preethi Honavar, S. Jotheeswaran, Meeka Khanna, Subasree Ramakrishnan

**Affiliations:** 1 Department of Neurological Rehabilitation, National Institute of Mental Health and Neurosciences (NIMHANS), Hosur Road, Bangalore, 560029 India; 2 Department of Neurology, National Institute of Mental Health and Neurosciences (NIMHANS), Bangalore, India

**Keywords:** Stroke, Overground gait training, MARS robot, Gait parameters

## Abstract

**Objective:**

To observe the effect of overground gait training with ‘Mobility Assisted Robotic System-MARS’ on gait parameters in patients with stroke.

**Patients & methods:**

This prospective pre-post study was conducted in a tertiary teaching research hospital with 29 adult stroke patients, with age up to 65 years. Patients fulfilling the inclusion criteria were divided in 2 groups based on the duration of stroke (≤ 6 months-sub-acute & > 6 months-chronic stroke) and provided overground gait training with MARS robot for 12 sessions (1 h/session) over a period of 2–3 weeks. Primary outcome measures were; 10-Meter walk test-10MWT, 6-min’ walk test-6MWT and Timed up & Go-TUG tests. Secondary outcome measures were Functional Ambulation Category-FAC, Modified Rankin Scale-MRS and Scandinavian Stroke Scale-SSS.

**Results:**

No adverse events were reported. Twenty-five patients who were able to perform 10-MWT at the beginning of study were included in the final analysis with 12 in sub-acute and 13 in chronic stroke group. All primary and secondary outcome measures showed significant improvement in gait parameters at the end of the training (*p* < 0.05) barring 10-Meter walk test in sub-acute stroke group (*p* = 0.255). Chronic stroke group showed significant minimum clinically important difference-MCID difference in endurance (6MWT) at the end of the training and both groups showed better ‘minimal detectable change-MDC’ in balance (TUG) at the end of the training.

**Conclusions:**

Patients in both the groups showed significant improvement in walking speed, endurance, balance and independence at the end of the training with overground gait training with MARS Robot.

**Clinical trial registry:**

National Clinical Trial Registry of India (CTRI/2021/08/035695,16/08/2021).

## Introduction

Stroke is the second leading cause of mortality, comprising 11.8% of all deaths worldwide, and the third most common cause of combined disability and death worldwide [1]. Locomotor disability is one of the significant barriers to community ambulation in stroke survivors and may manifest as reduced gait speed and endurance, recurrent falls, poor balance, and difficulty to perform activities of daily living [2]. Hence, recovery of gait is considered a top priority in rehabilitation of individuals with stroke.

In the acute phase of recovery, frequent, intensive, repetitive and task-specific training with active patient participation has been proposed to enhance neuroplasticity that facilitates gait and functional recovery [3, 4]. In recent years, stroke rehabilitation programs have incorporated use of several robotic devices, which provide more intensive and repetitive training compared to conventional approaches. A common characteristic of gait training robot is to partially support the body weight and aid in locomotion. Robotic devices can facilitate early mobilization of non-ambulatory patients and improve outcomes in the sub-acute phase of stroke [5]. The other advantages of robotic devices are their ability to deliver high repetitions of intensive gait training with reduced effort of the therapist, less energy-consumption, and greater cardiorespiratory efficiency of the patient. Treadmill-based robotics includes both end-effector devices and exoskeleton systems, which executes gait training on a treadmill with body weight support. In end-effector devices (e.g., G-EO- Reha-Technology, Switzerland), moveable footplates attached to the patient's feet simulate gait pattern. The exoskeleton treadmill system (e.g., Lokomat, Walkbot) moves joints, such as the hip, knee, and ankle, in a controlled manner during the gait training [6].

A systematic review suggested that patients who receive robotic-assisted treadmill gait training and physiotherapy after stroke might attain more independent walking than patients who receive only conventional training [5]. However, there was no difference in gait speed and endurance between robotic and conventional gait training with equal intensity and duration [5, 7, 8].

Despite the effectiveness of robot-assisted treadmill training, overground gait training is required to transfer the acquired skills to practical use in patients, improving the gait speed and endurance. Robotic Treadmill training does not permit the patient to experience real-world gait obstacles, such as walking on uneven terrain, stepping over objects, and stair climbing. Moreover, on treadmill robotics, patients walk with a pre-set speed and body weight support, creating an atmosphere where the patient might have less control in initiating each step and lack of alteration in visuospatial flow. These elements challenge optimum overground walking [9]. Therefore, stroke patients need to put more active effort into generating steps to walk and maintaining balance with the help or supervision during overground gait training. Traditionally, overground walking training is conducted using lower limb orthosis, walking aids such as cane/walker/hemiwalker etc., and therapists' assistance [10]. However, due to increased need of stroke patients and dearth of human resources including physical therapists, providing intensive and task-specific repetitive gait training is challenging [11]. Over-ground robotic-assisted gait training allows the patient to walk in a real-world setting, facilitates upright posture and balance control, and demands the patient's active participation while ensuring proper task performance [12]. Therefore, a robotic device using body weight support for overground walking could be a valuable tool for the gait rehabilitation of patients with stroke.

Overground robotic devices incorporate wearable powered exoskeletons (e.g. Ekso). Patients with severe deficits, including dense hemiplegia, might be benefited with exoskeleton robotic training [12]. The disadvantage is carrying the power source's heavy weight on the patient's back. Moreover patients with poor trunk control find it difficult to perform overground walking.

The Mobility Assisted Robotic System-MARS used in the present trial is an overground gait training-assist robot, developed by Bionic Yantra, an Indian start up based in Bengaluru, India. The system can sense the movement of the patient through the harness system, which is integrated with the sensor feedback system, allowing the patient to practice gait training safely and independently. There is a paucity of literature on robotic overground gait training in stroke patients. This pre-post study was aimed to explore the clinical effects of overground walking training with Mobility Assisted Robotic System (MARS) on gait parameters in stroke patients.

## Materials and methods

This prospective study was designed to evaluate the gait parameters in patients with stroke with MARS. We strictly adhered to ‘consolidated standards of reporting trials for non-pharmacological treatment (CONSORT-NPT)’ guidelines for reporting of this trial. The study was conducted in a neuro-rehabilitation unit at a tertiary care teaching hospital with stroke patients who were admitted for rehabilitation. The study was approved by the Institute Ethics Committee (NIMH/DO/IEC (BS&NS DIV)/2021–22 dated 18^th^ May 2021). The trial was registered with the National Clinical Trial Registry of India (CTRI/2021/08/035695).

### Participants

**Fig. 1:**
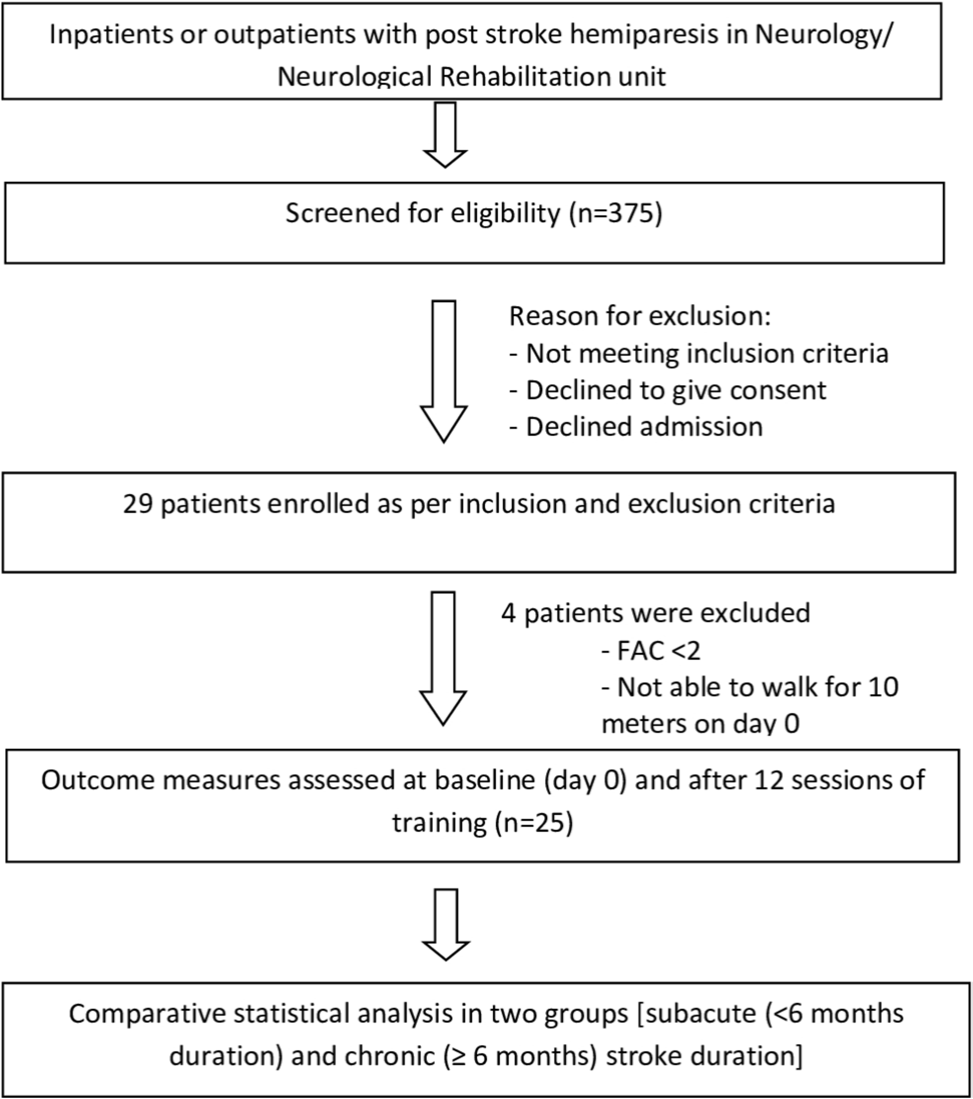
Flowchart of the study

Screening was done in the outpatient clinic of the department and patients with first ever stroke episode only were considered for the study. Those who gave written informed consent and satisfying the following inclusion criteria were recruited: (a) Adults with hemiparesis due to a first-ever arterial stroke with duration of ≥ 3 weeks post stroke, (b) Aged between 18 to 65 years, (c) Able to walk independently or with assistive device for 10 m, (d) Able to walk for 6 min at the beginning of the study and (e) Cognitive ability to consent, assimilate, and participate actively in the treatment protocol (Montreal Cognitive Assessment-MoCA ≥ 24). Individuals with bilateral motor deficits, global/Wernicke's aphasia, contracture of joints (hip, knee, and ankle) that would prevent from standing, walking, or fitting of harness system, recent Unstable Angina or Arrhythmia, any other medical conditions that prohibit intensive gait training, open skin ulcerations in the sacral / trochanteric region or other body surfaces in contact with harness affecting participation were excluded. Flow-diagram of the study is depicted in Fig. 1

### Intervention

#### Robotic device

**Fig. 2:**
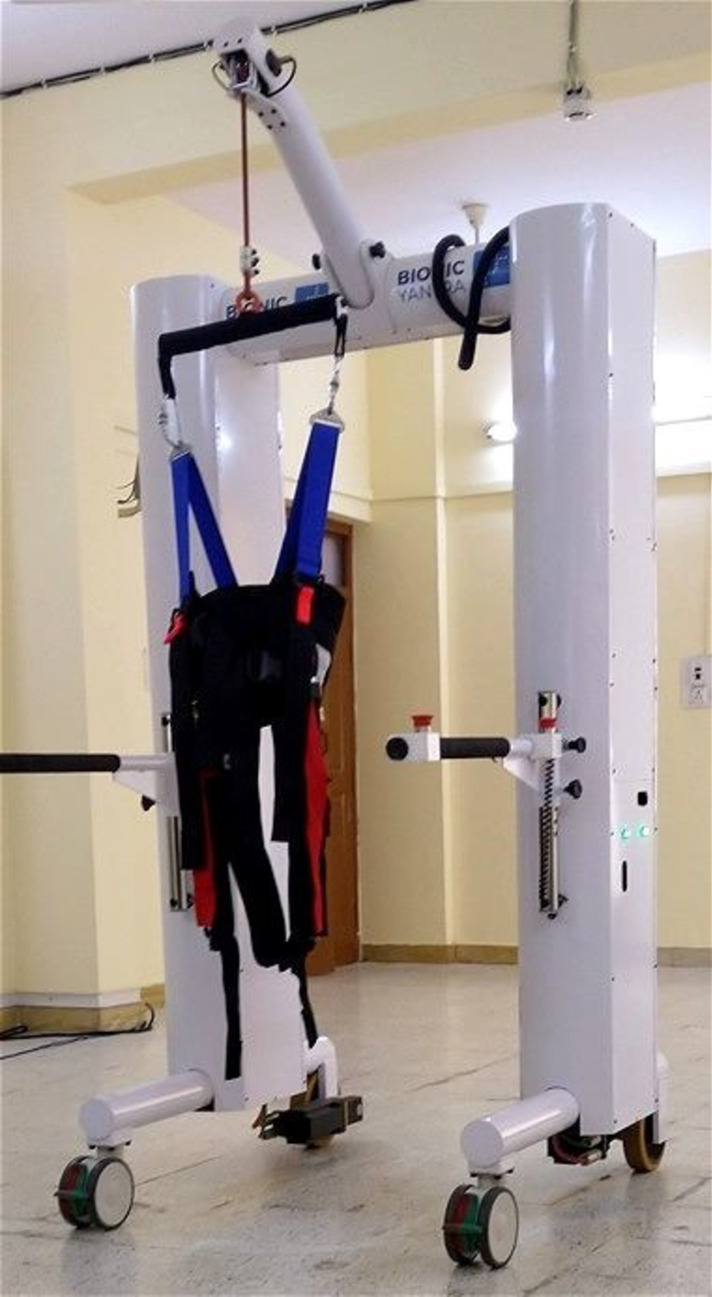
Device- mobility assisted robotic system

Mobility Assisted Robotic System (MARS) is a 'mobile robot' for overground walking and balance training, which is shown in Fig. 2. The MARS structure consists of an inverted U shaped frame approximately 2 m tall with powered wheels for forward and backward movement and castors for turning. The spreader bar and the harness system are suspended in the center, which attach to the patient through straps. Once strapped, the patient can be lifted from and back to a seated position using the winch motors. The "dynamic body weight unloading" or "Tension Control" is a feature of MARS, wherein a specific portion of the patient's body weight can be unloaded using the winch and held constant during the entire duration of the experiment. This unloading reduces the stress on the patients' lower limbs during standing or walking. Sensors in the system constantly monitor the centre of gravity, patient's load, position, acceleration, and intent to move. Any sign of fall is immediately detected and arrested. The robotic MARS system can move along with the patient without external intervention.

### Training protocol

Each patient underwent 5–6 sessions per week for a total of 12 gait training sessions over a period of ≥ 2 weeks. A zero session was conducted by an experienced physiotherapist (one of the co-authors) who had training with handling the device and the patients at the beginning of the study for familiarization with the device. The therapist checked for comfortable fitting of the harness for the body weight support before initiating step-by-step movement. A tuning exercise would be performed to determine the appropriate level of unloading and walking speed. The patient was trained to walk for a maximum duration of 1 h including resting period/s in between whenever required, depending upon the ability of the patient to use the body weight support system. The amount of unloading during training sessions varied from 20 to 0% of the body weight. It was based on the individual alignment of trunk and limbs with good weight shift and weight bearing onto the hemiplegic limb during the loading phases of gait. Progression of the gait training was achieved by reducing the body weight support, increasing the gait speed, and reducing the patient support from the handrail. Adverse Events, if any, were noted (like falls, pain, skin issues, device malfunctions etc.).

### Outcome measures

Outcomes were recorded at baseline (pre-training) and at the end of 12 training sessions (post-training). Primary outcome measures included; the 10-m walk test (10MWT) to assess walking speed expressed in meter/second [13] the 6-min walk test (6MWT) as a test of aerobic capacity/endurance measured as distance covered during the 6 min walking, expressed in meter [14]. The Timed Up and Go test (TUG)—time taken to get up from the chair, walk 3 m and return to sit on the chair to assess mobility and balance [15]. The secondary measures used were:; Functional ambulation Category (FAC) which assesses the level of assistance/dependence and supervision required for walking by the stroke subject [16] Modified Rankin Scale (MRS) to evaluate the degree of disability/ dependence after stroke [17]; Scandinavian Stroke Scale (SSS) to assess neurological impairment following a stroke [18].

### Data analysis

Data were analyzed using the Statistical Package for the social science statistical package SPSS version 22.0 (IBM, IL, Chicago, USA). Descriptive statistics included frequency, mean, median and standard deviation for quantitative variables such as age, gender and duration of stroke. As the data was ordinal with relatively small sample size of the study, non-parametric test Wilcoxon Signed rank test was applied to compare ‘pre versus post’ training mean scores for all outcome measures. The data was analysed in both the groups (sub-acute & chronic) separately. *P* < 0.05 was taken as the level of statistical significance.

## Results

Twenty-nine participants completed the MARS training protocol between April 2021 and July 2022. Four of them were not able to perform ‘10 m walk test’ on day 0 so they were excluded from the final analysis. Twenty-five patients who were included in the final analysis were stratified into two groups based on the stroke duration; sub-acute (≥ 3 weeks to ≤ 6 month) and chronic stroke (> 6 month). The demographic characteristics of all participants are enumerated in Table 1. There were 12 sub-acute and 13 chronic stroke patients with a mean age of 39.42 (SD 12.68) and 44.92 (SD11.24) years, respectively. The majority of the patients were males in the sub-acute group. Most participants had an ischemic stroke, and the mean time after stroke was 2.7 and 17.7 months in sub-acute and chronic stroke group, respectively.

**Table 1:** Baseline characteristics of sub-acute and chronic stroke patients

Characteristic	Sub acute stroke *n* = 12	Chronic stroke *n* = 13
Age (years), Mean ± SD	39.42 ± 12.68	44.92 ± 11.24
Sex (Male/female)	9/3	7/6
Type of stroke (Ischemic/haemorrhagic)	10/2	9/4
Side of hemiparesis (right/left)	6/6	7/6
Months post- stroke, Mean(SD)	2.7(1.4)	17.7(10.9)

All patients completed overground robotics training with MARS and no adverse events were reported. There were statistically significant improvements (*p* < 0.05) in mean gait speed, endurance among the chronic stroke group following overground robotic gait training compared to the baseline. Participants with sub-acute stroke also improved walking endurance (*p* = 0.001 with 6 MWT, *p* = 0.003 with 2 MWT) but no significant difference in gait speed after training (*p* = 0.255). Statistically significant improvement (*p* = 0.001) in balance post training was observed in both the groups (Table 2).

**Table 2:** Comparison of primary outcome measures before and after intervention in both groups

Variable	Sub acute stroke *n* = 12, mean ± SD			Chronic stroke *n* = 13, mean ± SD		
	**Pre-test**	**Post-test**	***p*-value**	**Pre-test**	**Post-test**	***p*-value**
**10MWT (m/sec)**	0.51 ± 0.21	0.57 ± 0.27	0.255	0.54 ± 0.26	0.69 ± 0.28	**0.002**
**6MWT (m)**	162.04 ± 73.4	193.54 ± 91	**0.001**	166.19 ± 80.8	203 ± 77	**0 .001**
**2MWT (m)**	53.75 ± 21.2	64.54 ± 27.7	**0.003**	57.31 ± 26.6	70.77 ± 25.9	**0.002**
**TUG**	31.26 ± 14.3	24.22 ± 11.9	**0 .001**	33.05 ± 20.5	25.26 ± 15.8	**0 .001**

10MWT = 10 Meter walk test, 6MWT = 6 min walk test, 2MWT = 2 min walk test, TUG = Timed Up and Go test

Changes in walking ability and function were evaluated using FAC. Table 3 shows the secondary outcome measures for both groups. There was statistically significant change in FAC in both groups (*p* = 0.011 & 0.018 respectively). Notably, the patients with sub-acute had a lower baseline FAC score than those with chronic stroke patients. Most patients had MRS grade 3 at the recruitment in the study and a statistically significant improvement was observed post intervention among sub-acute stroke patients (*p* = 0.037). Scandinavian stroke scale (SSS), which measures the impairment post-stroke showed significant changes in both groups at the end of the study (*p* = 0.009 & 0.004 respectively).

**Table 3:** Comparison of secondary outcome measures before and after intervention in both groups

Variable	Sub acute stroke *n* = 12, Median(IQR)			Chronic stroke *n* = 13, Median(IQR)		
	**Pre-test**	**Post-test**	***p*-value**	**Pre-test**	**Post-test**	***p*-value**
**Functional Ambulation Category (FAC)**	3.5(1.25)	4(1)	**0.011**	4(1)	4(1)	**0.018**
**Modified Rankin Scale (MRS)**	3(0)	2.5(1)	**0.037**	3(0)	3(1)	0.371
**Scandinavian Stroke Scale (SSS)**	46(10.5)	48.5(5.5)	**0.009**	44(6)	47(5)	**0.004**

The difference in scores of primary outcomes was calculated for each participant. The mean and median change of the above scores in both groups was computed. The pre-post score change and MCID (Minimal clinically important difference) and MDC (Minimum Detectable Change) are tabulated in Table 4.

**Table 4:** The primary outcomes scores (pre and post intervention). [MCID (Minimal clinically important difference) and MDC (minimal detectable change)]

Test	Difference in score pre-post robotic training				MCID
	**Sub-acute stroke**		**Chronic stroke**		
	**Median change**	**Mean change**	**Median change**	**Mean change**	
**10MWT (m/s)**	0.07	0.06	0.12	0.15	0.16
**6MWT (m)**	18.5	31.5	41	36.8	34.4
**2MWT (m)**	4	10.79	11	13.46	-
**TUG (s)**	8.4	7.04	5.7	7.79	2.9 (MDC)

10MWT = 10 Meter walk test, 6MWT = 6 min walk test, 2MWT = 2 min walk test, TUG = Timed Up and Go test

## Discussion

Robotic overground gait training is usually recommended to improve gait parameters in stroke patients. However, there is no consensus on the training frequency, duration, and chronicity of stroke patients who could get maximal benefit [19]. Current concepts tend to emphasize task-specific, high-intensity, repetitive rehabilitation strategies with early multisensory stimulation for motor learning [12]. Such features are characteristic of overground robot-assisted gait training, which offer more realistic task-specific and goal-oriented walking training and increase proprioceptive inputs than treadmill-based devices [20]. Literature is scarce regarding the overground robot-assisted gait training and further research about the robotic devices and gait training protocols is the need of the hour [21–24]. This study was an interventional pre-post study to assess gait parameters and clinical effects of overground gait training with the mobility-assisted robot in sub-acute and chronic stroke patients.

### Sub-acute group

Mean gait speed during spontaneous walking in the 10MWT increased from 0.51 m/s at baseline to 0.57 m/s at the end of the robotic training sessions, which was not statistically significant. This pre-post difference of 0.06 m/s is lower than the Minimally clinically important difference (MCID) of 0.16 m/s for acute stroke patients [25]. A few recent studies on overground gait training with the exoskeleton among sub-acute stroke reported contrasting results regarding gait speed [12, 21]. The characteristics of the study participants and the type of robotic device can probably explain this discrepancy. First, Calabro et al. (2018) and Gofferdo et al. (2019) did not use body-weight supported overground training, rather participants wore a commercially available powered exoskeleton [12, 21]. Individuals in our study had a mean walking speed of 0.51 m/s at baseline, whereas ambulant participants in the studies of Gofferdo et al. walked at a speed of 0.31 m/s at baseline [12]. In the present study, a higher baseline walking speed may have yielded a ceiling effect on the change in speed following training.

Statistically significant improvement was noted in 6MWT in sub-acute patients. The mean distance covered in 6 min increased from 162 m to 193.5 m at the end of the training. This mean pre-post difference of 31.5 m is less than the minimum clinically important difference-MCID of 34.5 m for 6MWT [26]. The TUG test is a valid and excellent tool for evaluating balance and mobility in stroke patients [27]. We observed both a statistically significant reduction in time by 7 s at the end of the study as well as better scores than MDC (2.9 s) after robotic training. These findings were in agreement with some recent studies [12, 21]. The median FAC surpassed from category level 3 to 4. So patients reached from ambulator-dependent level to ambulator-independent level. Again the result is in concurrence with the earlier studies on the use of treadmill robotic and overground exoskeleton robotic training [12, 28–30]. Improvement in FAC score is meaningful as it can predict independent community ambulation six months after stroke [5].

### Chronic stroke group

A statistically significant change in 10MWT, 6 MWT, TUG, and FAC scores was observed with robotic training. Average gait speed increased from 0.54 m/s at baseline to 0.65 m/s, and the distance covered in 6 min increased from 166 to 203 m following training. The mean improvements in speed and endurance were 0.15 m/s and 37 m, respectively, this is higher than the MCID in chronic stroke patients (0.14 m/s for velocity, 34.5 m for 6MWT) [25, 26].

Literature reports mixed observations regarding walking speed in chronic stroke patients before and after body-weight supported robotic treadmill training [5, 7, 8, 31, 32]. Factors that need to be considered are treadmill-based robotic training allows motions only in the sagittal plane. Although treadmill and body weight support can deliver high-intensity gait training, it may not be task specific to overground walking in natural setting [5].

A recent review reported that exoskeleton overground gait training is analogous to conventional training for chronic stroke patients [24]. Tedla et al. (2019) reported no additional effect on gait speed in the subacute phase but a positive trend in the chronic phase, which is in concurrence with our study results [7]. Another recent study reported positive outcomes of robotic overground gait training in both sub-acute and chronic stroke patients [22]. In our study, all the patients underwent robotic overground gait training without any traditional gait training; hence, their gains in gait speed, 6MWT, TUG, and FAC can be attributed to the robotic treatment. Although a randomized-control trial with sham group would provide more definitive answers. The improvement in gait parameters with MARS could be explained by the fact that the intervention provides the opportunity to perform more intensive, repetitive, and task-oriented active training in real-life (overground) situations.

### Direction for future research

The present study aimed to study the safety and efficacy of a novel overground robotic gait trainer in patients with stroke. A controlled trial, larger sample size and further stratification with regard to age, associated sensory deficits etc. would be the way forward to establish the advantage of this device over the other methods and contraptions for gait training. Longitudinal study would help in looking for the sustainability of gains made by training with the MARS. Instrumental gait analysis may give information regarding the impact of overground robotic training on electromyographic activation patterns while training with MARS.

## Conclusions

This study demonstrates that overground gait training with MARS, when executed by ambulatory stroke patients is safe. Patients showed significant improvement in walking speed, balance, endurance and independence at the end of the training. Further studies may look into the predictors influencing the improvement of gait parameters following overground robotic training.

## Data Availability

We have the data available with us which can be shared on request. The contact person for such a request would be AG.
